# Osteopontin activates mesenchymal stem cells to repair skin wound

**DOI:** 10.1371/journal.pone.0185346

**Published:** 2017-09-28

**Authors:** Wenping Wang, Pei Li, Wei Li, Junzi Jiang, Yanyan Cui, Shirong Li, Zhenxiang Wang

**Affiliations:** 1 Department of Plastic and Aesthetic Surgery, Southwest Hospital, Third Military Medical University, Chongqing, China; 2 Department of Orthopedics, No.89 Hospital of People’s Liberation Army, Weifang, Shandong, China; 3 Department of Plastic and Burn Surgery, The First Affiliated Hospital of Bengbu Medical College, Bengbu, China; 4 Department of Genetics and Cell Biology, Chongqing Medical University, Chongqing, China; Northwestern University, UNITED STATES

## Abstract

Mesenchymal stem cells (MSCs) are promising candidates for skin wound repair due to their capabilities of accumulating at wounds and differentiating into multiple types of skin cells. However, the underlying mechanisms responsible for these processes remain unclear. In this study, we found that osteopontin (OPN) stimulated the migration of MSCs in vitro, and observed the recruitment of endogenous MSCs to a skin wound and their differentiation into keratinocytes and endothelial cells. In OPN knock-out mice, the recruitment of MSCs to the skin wound was significantly inhibited, and wound closure was hampered after an intradermal injection of exogenous MSCs compared to wild-type mice. Consistent with these observations, the expressions of adhesion molecule CD44 and its receptor E-selectin were significantly decreased in the lesions of OPN knock-out mice compared with wild-type mice suggesting that OPN may regulate the migration of MSCs through its interactions with CD44 during skin wound recovery. In summary, our data demonstrated that OPN played a critical role in activating the migration of MSCs to injured sites and their differentiation into specific skin cell types during skin wound healing.

## Introduction

Skin wound healing is a multi-stage process that orchestrates the reconstruction of dermal and epidermal layers. This process involves three overlapping phases, including the inflammatory, proliferation, and remodeling phases. Mesenchymal stem cells (MSCs) can differentiate into a variety of cell types, including osteoblasts, chondrocytes, adipocytes, myoblasts[1] endothelial cells[2, 3], keratinocytes[2] neural cells[4, 5], and hepatocytes[6, 7] in vitro. In vitro, MSCs can differentiate into tissue-specific cells in response to cues provided by different organs[8] MSCs could differentiate to endothelial cells, myofibroblasts and pericytes cells, promoting wound healing in vivo[2]. In addition, MSCs are also characterized by immunosuppressive effects on the surrounding environment after transplantation[9, 10]. MSCs have been used in clinical trials[11, 12]for the successful treatment of chronic wounds[13] MSCs are reported to be involved in all three phases[14–16]of skin wound healing.

Osteopontin (OPN) is a glycosylated phosphoprotein. It can be found in body fluids and the extracellular matrix of mineralized tissues[17].OPN responds to various stimulations such as inflammation, cellular stress, and injury and its expression increases during tumorigenesis and angiogenesis[18–22]. OPN can activate various signal pathways and modulate cellular activities[17, 23]by binding and interacting with specific cell surface receptors, including integrin and CD44 receptor variants[17, 24].OPN can regulate cell migration, extracellular matrix (ECM) invasion, and cell adhesion in endothelial and epithelial cells through interactions with cell surface receptors[23, 25] OPN also plays a key role in the regulation of tissue remodeling[17]. It has been shown that the expression of OPN increases during wound healing, compared to healthy skin[26]. OPN knock-out (*Opn*^-/-^) mice show alterations in wound healing, including matrix disarrangement and modifications of collagen fibrillogenesis in the cartilage compared with their wild-type littermates[26].

A previous study found that OPN expression was significantly up-regulated during wound healing, but little is known about its mechanisms[27]This paper shows that the expression of osteopontin increases in wounds, facilitating the mobilization of bone marrow derived stem cells into wounds by interacting with CD44. The accumulated MSCs at wound sites subsequently transdifferentiates into multiple skin cell types and contribute to wound repair.

## Materials and methods

### Ethical statement

*Opn*^-/-^ mice were purchased from The Jackson Laboratory (USA, Stock No: 004936). Mice were bred in the specific pathogen free (SPF) unit Experimental Animal Center, Daping Hospital, Third Military Medical University. All experimental procedures were approved by the Animal Ethics Committee of the Third Military Medical University, China. This study was carried out in strict accordance with the recommendations in the Guide for the Care and Use of Laboratory Animals of the National Institutes of Health. The protocol was approved by the Committee on the Ethics of Animal Experiments of the Third Military Medical University. All surgery was performed under sodium pentobarbital anesthesia, and all efforts were made to minimize suffering.

### Mice

*Opn*^-/-^ mice were purchased from The Jackson Laboratory (USA, Stock No: 004936). The mice were bred in the specific pathogen free (SPF) unit of the Experimental Animal Center, Daping Hospital, Third Military Medical University. All experimental procedures were approved by the Animal Ethics Committee of the Third Military Medical University.

### Isolation and culture of MSCs

MSCs were collected by flushing the femurs and tibias of new born *Opn*^-/-^ mice and wild-type C57BL/6 mice. These cells were cultured in bone marrow stromal cell (BMSC) basic medium containing MSC stimulatory supplements (Cyagen Biosciences, Santa Clara, CA, USA). After 24 h of incubation, the cell culture dishes were washed by PBS to remove floating cells and fresh medium was added. The cell culture medium was refreshed every 2 days. The adherent cells from the third passage were used for experiment[28].

### Flow cytometry

The cultured MSCs were washed in PBS, trypsinized, washed in PBS-5% FBS and incubated with FITC-conjugated anti-CD3(BD Biosciences,553733), CD29(BD Biosciences,55500), CD117(BD Biosciences,561680) or PE-conjugated anti-CD105(BD Biosciences,562759), CD44(BD Biosciences,561860) and Sca-1(BD Biosciences,553108) antibodies (10 μg/mL in PBS-0.5% bovine serum albumin) for 1 h, then washed 3 times with PBS. All incubations were performed at 4°C. MSCs, MSCs with FITC and PE dye without antibodies were used as controls. Lastly, all the cells were analyzed on a FACS can instrument (Attune® Acoustic Focusing Cytometer, Applied Biosystems)[28]

### Directed migration assay in vitro

The migration assay was performed using 24-well transwell chambers (8 μm, Millipore, Billerica, MA). The lower chambers contained 600 μl of serum-free medium with or without 25 nM/ml recombinant mouse OPN (R&DSystems, Minneapolis, MN). Approximately 2×10^4^ cells of wild-type and *Opn*^-/-^ MSCs in 100 μl serum-free medium were placed in the upper chamber. The plates were incubated at 37°C in 5% CO2. After 24 hours, methanol was used to immobilize the cells culturing on the transwell chambers and then 0.05% crystal violet was added to stain the cells. After staining for 15min, cotton-tipped swabs were used to remove the cells, and then the cells were washed through filters with PBS. A microscope was used to examine and count the cells on the bottom of the filters. We randomly chose four different fields from each insert and counted the number of cells in each field.

### Differentiation of the MSCs into the mesenchymal lineage

For MSC differentiation, a 70% subconfluent culture of MSCs from the third passage were used.

The MSCs were placed in basic medium, consisting of Dulbecco’s modified Eagle’s medium (DMEM) (Invitrogen Life Technologies), 10% fetal bovine serum (FBS), 1% penicillin, 1% streptomycin and 1% amphotericin B. Specific supplements were added to this basic medium formulation for the differentiation of the MSCs into various mesenchymal lineages[28]. Adipogenic differentiation was induced with 0.5 μM dexamethasone, 0.5 Mm 3-isobutyl-1-methylxanthine and 0.1 mM indomethacin (Sigma)[29]. Osteogenic differentiation was achieved with 0.1 μM of dexamethasone, 50 μM ascorbic acid and 10 mM-glycerophosphate (Sigma)[30]. Chondrogenic differentiation was induced with 50 μM ascorbic acid, 0.1 μM dexamethasone, 10 ng/ml transforming growth factor-beta (TGF-β) (R&D Systems), 40 μg/ml L-proline (Sigma- Aldrich) and 100 μg/ml sodium pyruvate (Wako) [31]. The differentiation medium was refreshed every 2 days. Differentiation into adipocytes, osteocytes and chondrocytes was confirmed by oil red O, von Kossa and toluidine blue staining, respectively[30]

### Differentiation of MSCs into keratinocytes

MSCs were cultured on 8-well glass slide chambers with keratinocyte basal medium (Invitrogen Life Technologies) containing 0.5 nM bone morphogenetic protein-4 (BMP-4) (SAB), 0.3 mM ascorbic acid and 3 ng/ml epidermal growth factor (Pepro Tech; Rocky Hill, U.S.A)[32]. The medium was changed every 2 days. After 17 days, the MSCs were stained with a keratin 14 (CK14) antibody (Abcam, ab7800, diluted 1:300).

### Endothelial cell differentiation

Confluent MSCs cells were cultivated in the presence of 4% FBS, 50 ng/ml vascular endothelial growth factor (VEGF) (Pepro Tech; Rocky Hill, U.S.A), 10 ng/ml basic fibroblast growth factor (b-FGF) (Pepro Tech; Rocky Hill, U.S.A), 100U/ml penicillin and 100U/ml streptomycin for 12 days[33, 34]. Then the MSCs were stained with a von Willebrand factor (vWF) antibody (Abcam, ab11713, diluted 1:500). The medium was changed every 2 days.

### In vitro angiogenesis

A capillary formation assay was performed strictly following the manufacturer’s instructions and examined with an in vitro angiogenesis kit (Chemicon; Temecula, CA). A 50 μl gel matrix solution was applied to one well of a 96-well plate and incubated for 1 hour at 37°C. The cells that differentiated into endothelial cells were then trypsinized, and 1x10^4^ cells were resuspended in 100 μl of the endothelial medium, plated onto the gel matrix and cultured for 18 hours. The percentage of capillaries formed was calculated from three independent experiments.

### Skin incisional wounding and a directed migration assay in vivo

Male wild-type C57BL/6 and *Opn*^-/-^ mice (8–10 weeks old) were anesthetized, and full-thickness punch biopsy wounds of 5 mm in diameter were made on the animals’ backs. A total of 5x10^5^ wild-type GFP MSCs were intradermally injected 5 mm from the wounds. And injection of phosphate-buffered saline (PBS) at the wound periphery was performed as a control. Standardized images of the wounds were taken with a digital camera for daily wound size analyses. The next day after the invasive operation, the migration of MSCs was observed with a small animal in vivo imaging instrument (Xenogen IVIS spectrum, caliper, USA) and fluorescence was evaluated daily until the 7th day. Skin samples were collected at specific time points after wounding. For immunofluorescence staining, the skin samples were fixed in 4% paraformaldehyde and embedded in paraffin. A statistical analysis was performed using Student's t-test, with n = 6 animals per group.

### Immunofluorescence staining

Skin sections were stained with an anti-GFP antibody. The skin sections were also treated with primary Abs against CD31 (Abcam, ab28364, diluted 1:300), vWF (Abcam, ab11713, diluted 1:300), CK14 (Abcam, ab7800, diluted 1:500), CD44 (Abcam, ab157107, diluted 1:400), E-selectin (Abcam, ab18981, diluted 1:400), Secondary antibodies conjugated to rhodamine-isothiocyanate were used for fluorescence detection on a confocal laser scanning fluorescence microscope (Zeiss, German).

## Statistical analysis

The data are expressed as the means ± SD of the indicated number of observations. In all instances in which a radioisotope was used, background radioactivity was subtracted before quantifying radioactivity. Comparisons between two groups were performed using the unpaired, two-tailed Student's t-test. If necessary, a one-way analysis of variance (ANOVA) was applied for comparisons among multiple groups. In all cases, P values less than 0.05 were considered significant.

## Results

### Characterization of isolated MSCs

**Fig 1:**
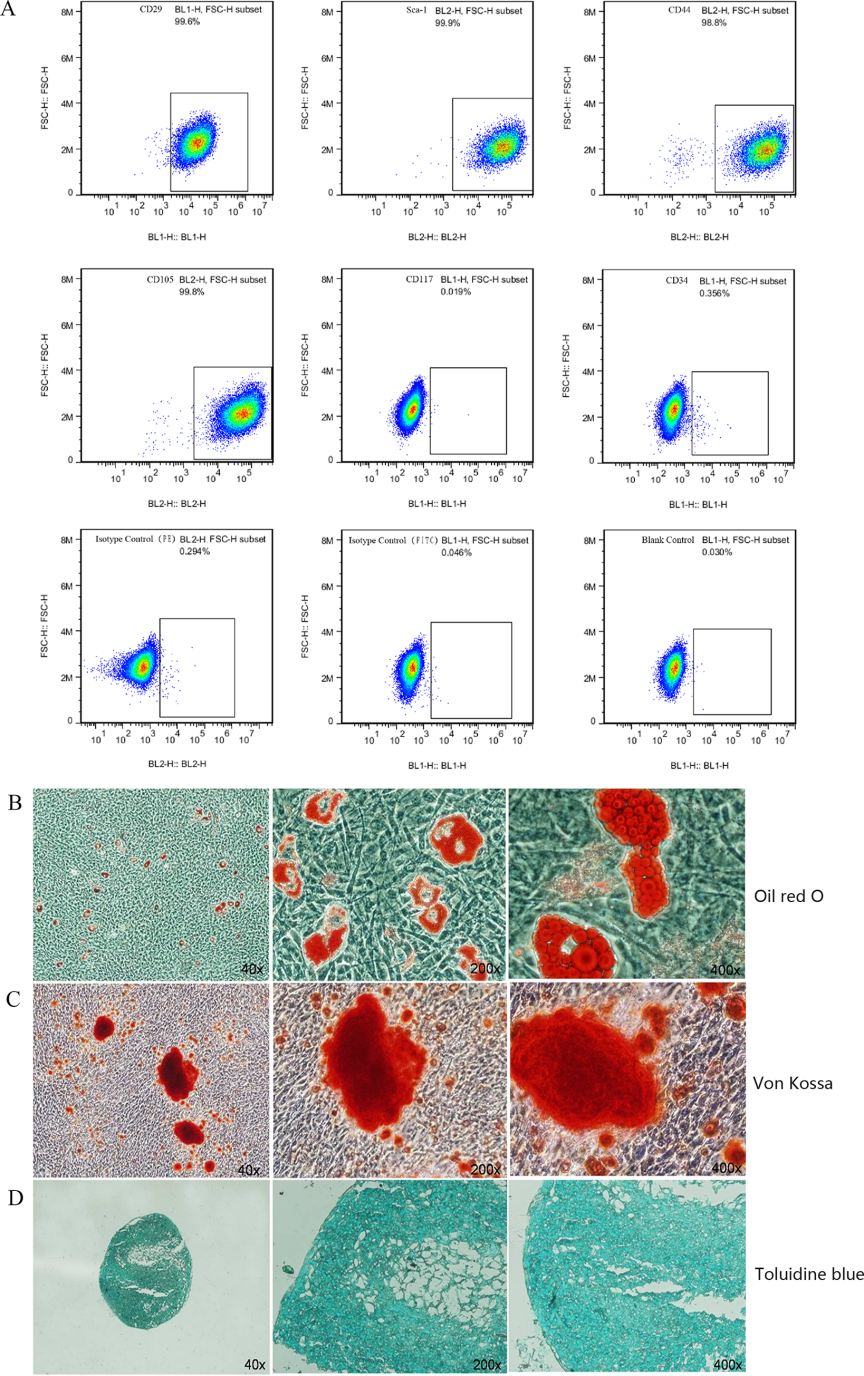
Isolated cells have the characteristics of MSCs. (A)Cell surface markers of MSCs were assessed by FACS. MSCs expressed CD29, Sca-1, CD44, and CD105, but not CD34 and CD117. The isotype controls and blank control showed negative results. (B)Adipogenic differentiation was revealed with oil red O staining. (C) Osteogenic differentiation was confirmed by von Kossa staining. (D) The chondorogenic potential of MSCs was determined by staining for toluidine blue.

To characterize the isolated MSCs, flow cytometry was performed to analyze the expressions of cell surface markers of MSCs. The expressions of CD29, CD44, CD105 and Sca-1, but not CD34 and CD117, were positive in MSCs (Fig 1A), which is consistent with previous reports[30, 35]. Moreover, MSCs were also evaluated for their differentiation ability to adipogenic, osteogenic, and chondrogenic cells. These cell types are positive for oil red O staining, von Kossa staining, and toluidine blue staining, indicating adipogenic, osteogenic, and chondrogenic cell type differentiation, respectively (Fig 1B–1D). Only the cells that were consistent with these criteria were selected for our experiments.

### Recombinant OPN promoted the migration of MSCs

**Fig 2:**
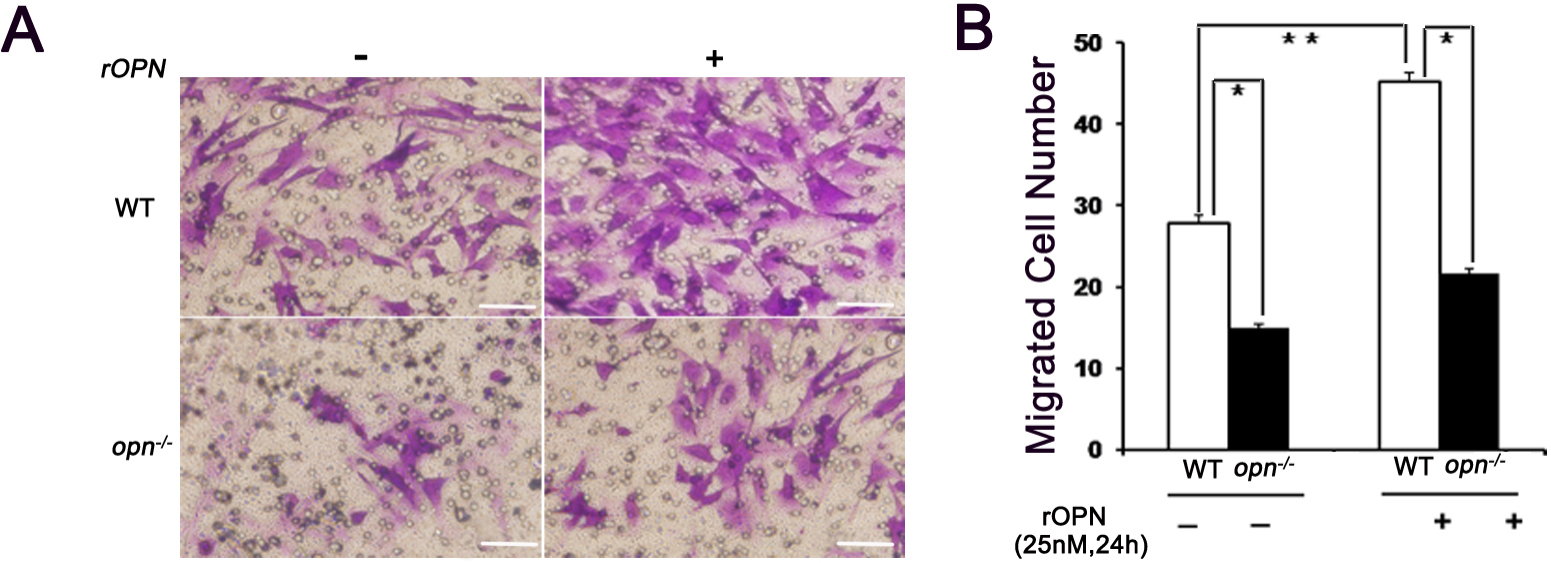
The effects of rOPN on the migration of MSCs. (A) MSCs were seeded on transwells with the presence of 25nM rOPN on the bottom. (B) After 24 hours, the number of cells present on the bottom side of the transwells’ filter was counted and analyzed. Scale bars indicate 50 μm. *and **p < 0.01(n = 4), determined by a one-way ANOVA.

The migration assay was performed using 24-well transwell chambers. The cells on the underside of the filters were detected and counted under a microscope 24 hours later. Images were taken randomly in four fields from each insert. The experiments showed that the number of migrated wild-type MSCs was greater than the number of migrated *Opn*^-/-^ MSCs, and that the addition of rOPN increased the migration potential of the MSCs (Fig 2).

### The differentiation of MSCs into endothelial cells and keratinocytes were OPN-dependent

**Fig 3:**
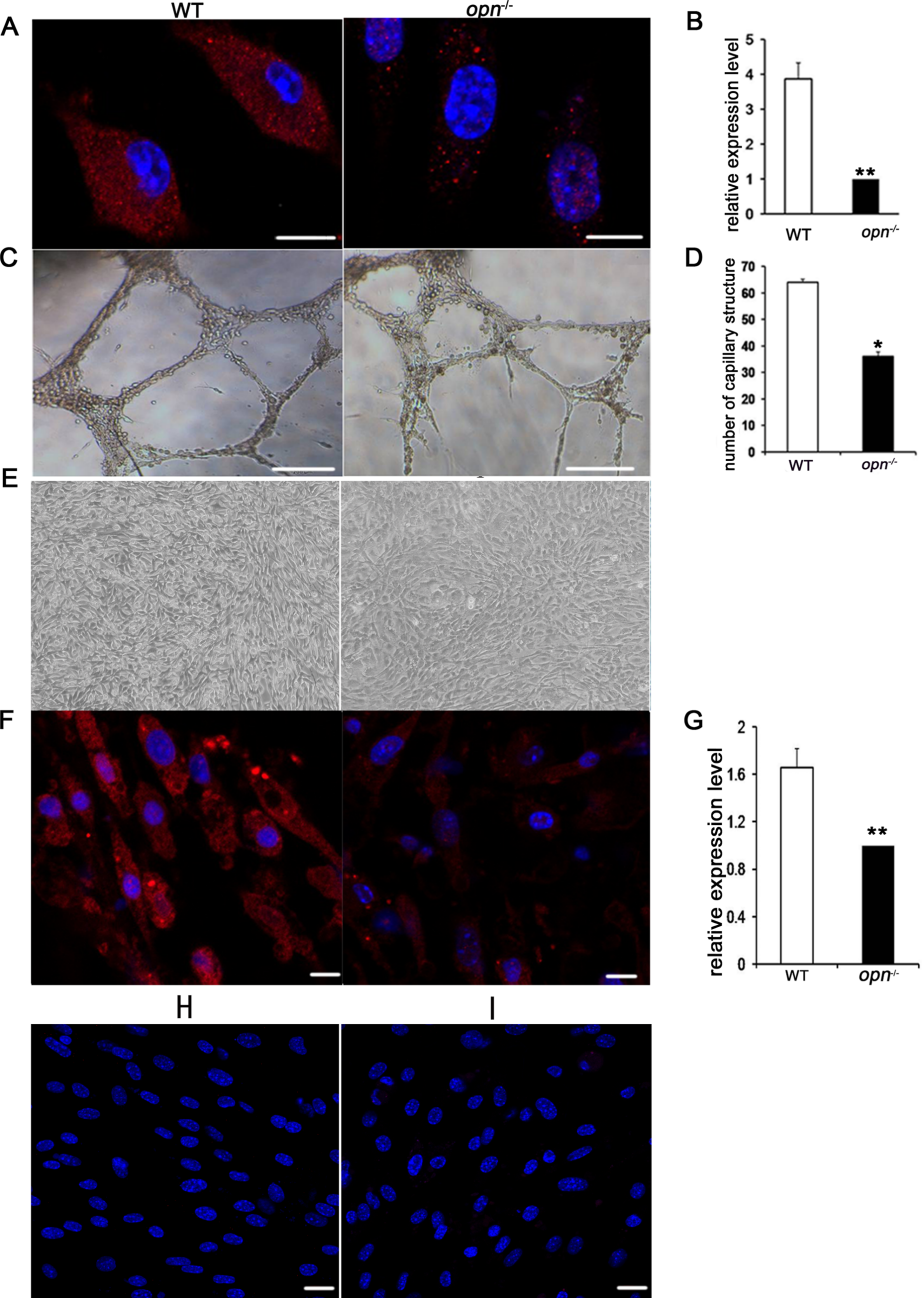
MSCs can differentiate into endothelial cells and keratinocytes in vitro. (A) von Willebrand staining of differentiated MSCs. Differentiated wild-type MSCs showed stronger signals. (B) Immunofluorescence analysis of von Willebrand in differentiated MSCs. (C) Analysis of capillary-like structures formation on Matrigel^TM^. Wild-type and *Opn*^*-/-*^ MSCs can form similar capillary-like structures on Matrigel^TM^. (D) Wild-type MSCs formed more capillary-like structures than *Opn*^*-/-*^ MSCs. (E) MSCs can differentiate into endothelial-like cells. (F) Keratin14 staining of differentiated MSCs. (G) Immunofluorescence analysis of keratin14 in differentiated MSCs. (H) Undifferentiated MSCs from wild-type mice were stained with Von Willebrand factor. (I) Undifferentiated MSCs from wild-type mice were stained with Keratin14. Scale bars indicate 200 μm in (A), (C) and 20μm in (F), (H) and (I), respectively. * *p* < 0.05 and **p < 0.01, (n = 3), determined by Student's t-test.

Endothelial cells and keratinocytes have very important roles in wound healing. To assess whether MSCs can trans-differentiate into these two cell types in vitro, wild-type and *Opn*^-/-^ MSCs were exposed to endothelial and keratinocyte induction systems. At day 12 after differentiation, the expression of vWF, an endothelial-specific marker, was detected by immunofluorescence, and the expression of vWF in wild-type MSCs was higher than in *Opn*^-/-^ MSCs (Fig 3A and 3B). The angiogenic capability of MSCs was assessed using an in vitro capillary formation assay. As shown in Fig 3C, capillary-like structures that originated from wild-type and *Opn*^-/-^ MSCs were similar, but their total tube numbers were significantly different (Fig 3D). In addition, at day 17 after epithelial induction, the morphology of some cells had changed into the typical pebble-like shapes of epithelial cells, losing their original spindle-like-shapes (Fig 3E). Furthermore, most of these pebble-like epithelial cells expressed the keratin 14 protein, an epithelial cell marker (Fig 3F and 3G).

### OPN regulated the migration of MSCs into wound sites

**Fig 4:**
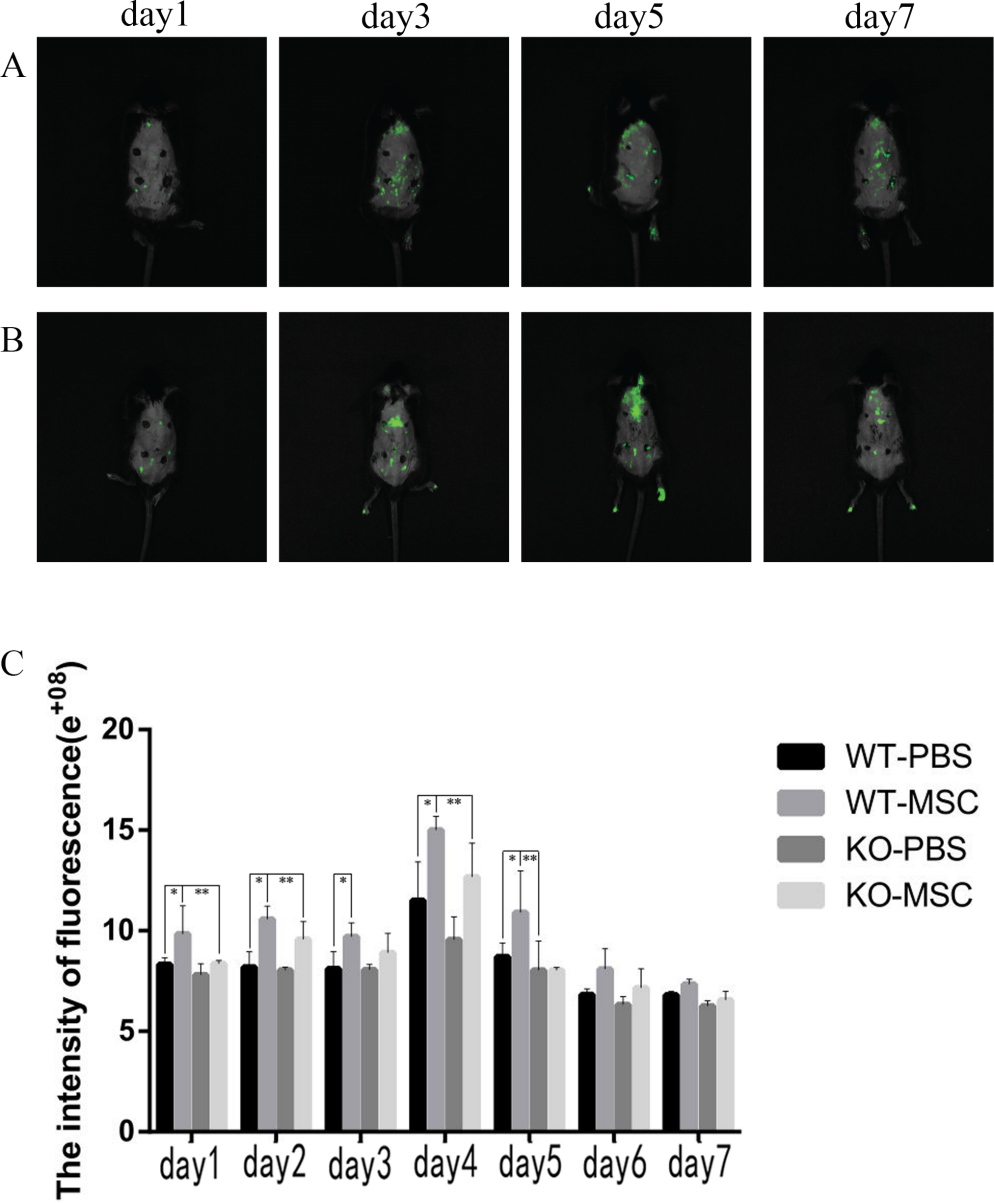
In vivo imaging of injected wild-type GFP MSCs. In vivo image tracking of injected wild-type GFP MSCs in live wild-type (A) and *Opn*^*-/-*^ mice (B). (C) Cells migration assay (the fluorescence intensity) of GFP MSCs from wild-type and *Opn*^*-/-*^ mice. * and **, *p* < 0.05, (n = 5), determined by a one-way ANOVA.

To evaluate OPN’s effect on the migration of MSCs, circular full-thickness wounds with a diameter of 5 mm were created on the backs of wild-type and *Opn*^*-/-*^mice. A total of 5×10^5^ wild-type GFP positive MSCs were injected directly into the wound site and 5 mm away from the wound, respectively. In vivo images were obtained from each mouse every day by the IVIS system. As shown in Fig 4, the GFP signal was detected from the first day after injection in wild-type mice and accumulated in the wounds from the third day on. Migration was greatest on the fifth day (Fig 4A and 4C). In contrast, in *Opn*^*-/-*^ mice, the GFP signal was obviously weaker, most likely because the cells had migrated elsewhere (Fig 4B and 4C).

### OPN leads to the differentiation of MSCs into multiple skin cell types

**Fig 5:**
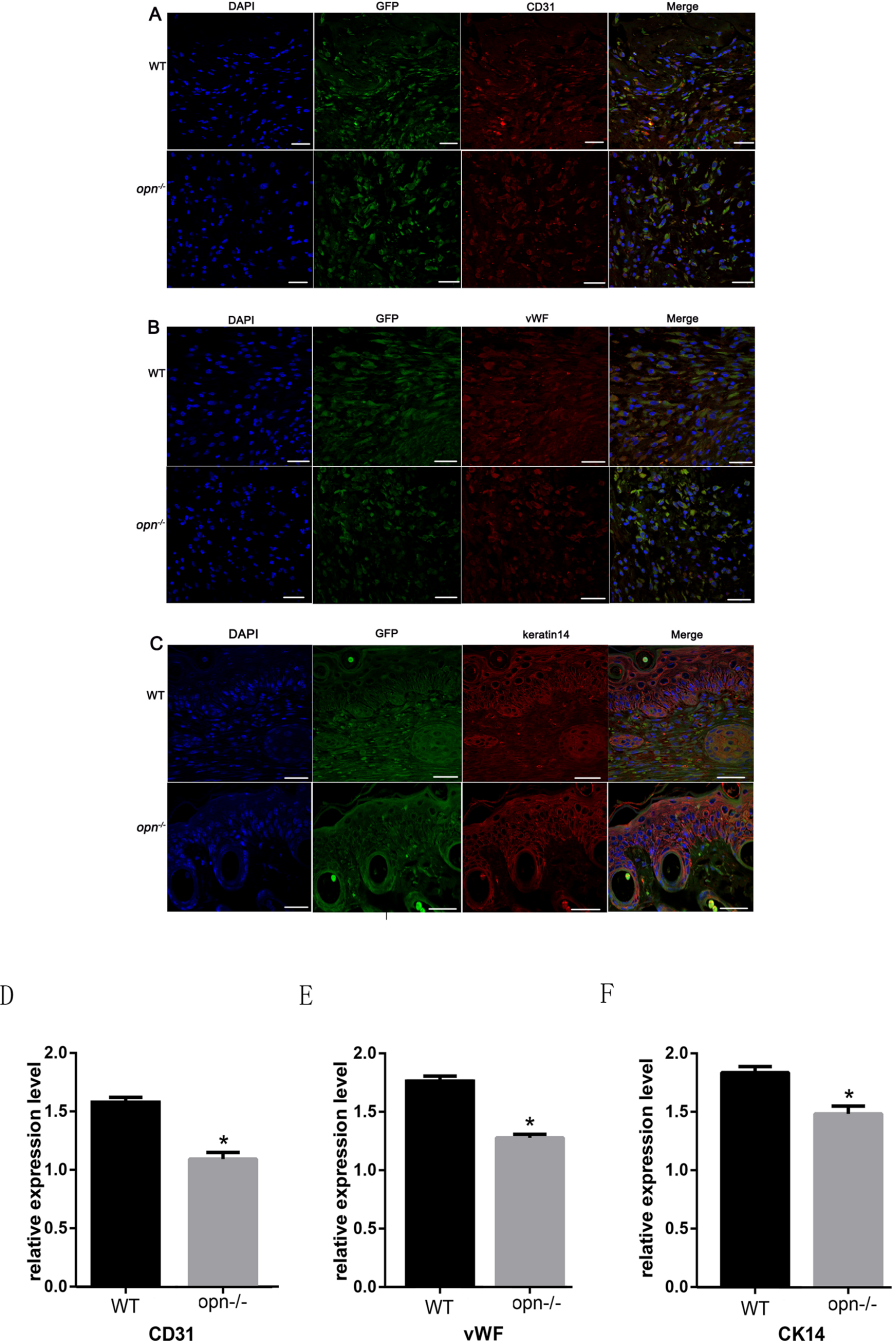
Immunofluorescence analysis of CD31, vWF and keratin 14 expressions in wound sites. (A) GFP positive cells (green) were colocalized with CD31 (red), (B)vWF (red) and (C)keratin 14. Nuclear staining with DAPI is blue. (D)(E)(F) Immunofluorescence analysis of CD31, vWF and keratin14 in wound sites. These data suggest that MSCs differentiated into endothelial cells and keratinocytes, respectively. Scale bars indicate 50 μm. * *p* < 0.01, (n = 5), determined by Student's t-test.

MSCs could differentiate into multiple skin cell types during wound healing[2]. To identify whether OPN regulates the differentiation of MSCs into skin cells during wound healing, wild-type GFP MSCs were injected into injured skin sites in wild-type and *Opn*^-/-^ mice. Wounded skin samples were harvested at indicated time points (the seventh day after injection) for immunofluorescence staining of skin cell markers. We found that GFP-positive cells were co-labeled with endothelial cell markers CD31 and vWF (Fig 5A and 5B) and keratinocyte marker keratin 14 (Fig 5C). However, the number of GFP-positive cells in the wild-type mice was greater than in the *Opn*^-/-^ mice, indicating that OPN recruited more MSCs to the wound sites in the wild-type mice. Moreover, the percentages of GFP and CD31, vWF or keratin 14 co-labeled cells were higher in the wild-type mice compared to the *Opn*^-/-^ mice (Fig 5D–5F). In addition, the expressions of CD31, vWF, and keratin 14 were higher in the wild-type mice compared to the *Opn*^-/-^ mice.

### OPN deficiency impedes wound healing in MSCs treatment of skin injury

**Fig 6:**
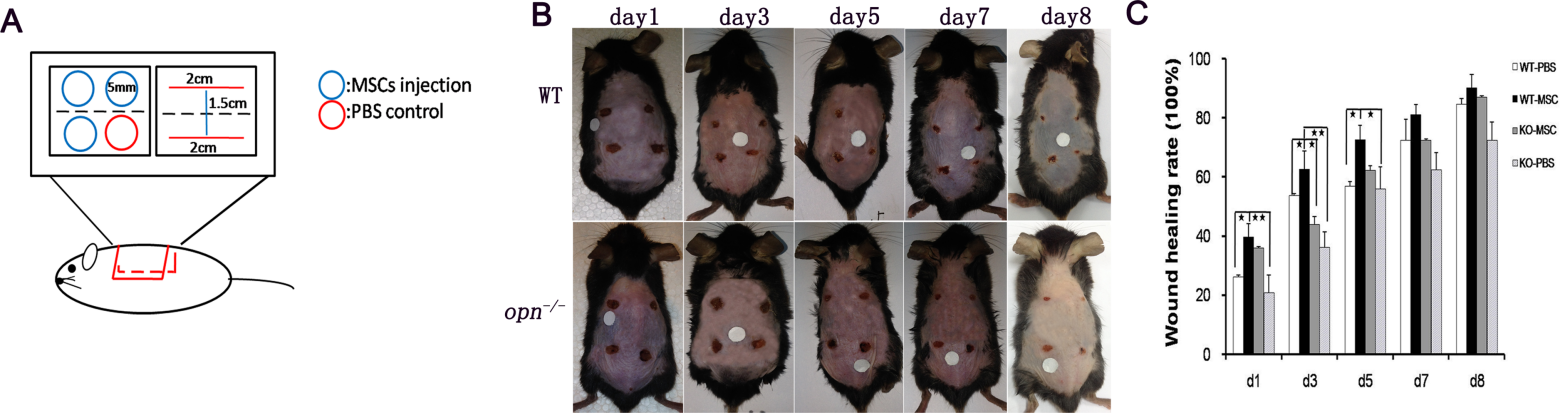
Skin wound healing analysis in wild-type and *Opn*^*-/-*^ mice. (A) A schematic diagram illustrating the location and dimensions of the full-thickness excisional and incisional wounds made to the shaved dorsal skin of adult male mice. The dotted lines indicate the axes of sections. Blue circles indicate MSCs injection and red represents PBS control injection. (B) Observation of wild-type and *Opn*^*-/-*^ wounds at various time points after wounding. (C) The proportion of wound healing relative to the initial wound area at each time point after injury in the control (open bars) versus *Opn*^*-/-*^ wounds. * *p* < 0.05 and **p < 0.01, (n = 6), determined by a one-way ANOVA.

We compared the wounds between wild-type and *Opn*^-/-^ mice during the repair process. As shown in Fig 6B and 6C, treatment with MSCs by injection accelerated wound healing in the wild-type mice, but similar treatment result was not observed in the *Opn*^-/-^ animals with the same MSCs administration. After MSCs treatment, the rate of wound healing in the wild-type mice was dramatically higher than in the *Opn*^-/-^ mice at early time points (Fig 6B and 6C), with a mean of 62.6% closure achieved by 3 d in the wild-type mice, which is significantly higher than 36.2% closure in the *Opn*^-/-^ mice. The rate of wound healing in the wild-type MSCs group was higher than in the *Opn*^*-/-*^ MSCs group at days 7 and 8, but there was no statistically significant difference between the two groups (Fig 6C), possibly because the number of animals in each group was relatively small (6 mice per group).

### CD44 and E-selectin expressions were down-regulated in Opn-/- mice

**Fig 7:**
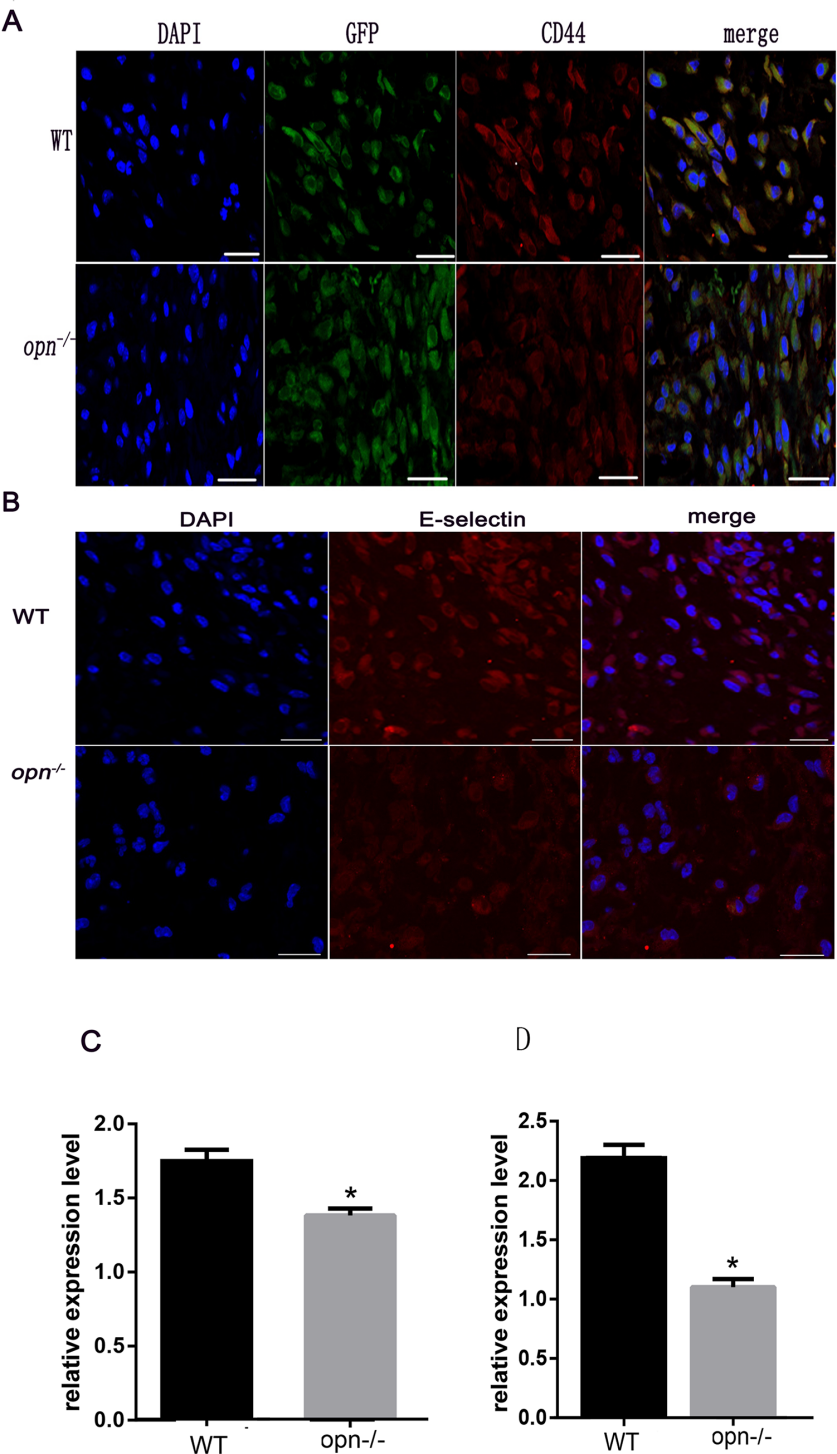
Immunofluorescence analysis of CD44 and E-selectin expressions after injury. (A) (B)GFP positive cells (green) colocalized with CD44 and E-selectin (red). Nuclear staining with DAPI is blue. (C) (D)Immunofluorescence analysis of CD44 and E-selectin in the wound sites. The CD44 and E-selectin signal are stronger in the wild-type mice. Scale bars indicate 50 μm. * *p* < 0.01, (n = 5), determined by Student's t-test.

MSCs constitutively express the cell surface receptor CD44, which can interact with OPN to increase cell motility, invasion and angiogenesis[36, 37] As shown in Fig 7A and 7C, immunofluorescence staining showed that CD44 expression was attenuated in *Opn*^*-/-*^ mice compared to wild-type mice, indicating that OPN/CD44 signaling may play a role in MSC-mediated wound healing.

CD44 is the ligand of E-selectin, which plays an important role in mediating tissue-specific T cells into skin and primitive hematopoietic progenitor cells (HPCs) into bone marrow (BM)[38].To explore whether CD44 and E-selectin are involved in OPN-mediated wound healing, the expression of E-selectin was detected. As shown in Fig 7B and 7D, E-selectin expression was attenuated in *Opn*^*-/-*^ mice compared with wild-type mice, suggesting that decreased E-selectin/CD44 expression was associated with OPN deficiency and contributed to the recruitment of MSCs to injured sites.

## Discussion

MSCs represent a powerful tool for skin wound therapy, but the underlying mechanism remains unknown. In this paper, we showed that OPN promoted the activation of MSCs, including their migration and specific cell differentiation during skin wound healing and in vitro. MSCs derived from both wild-type and *Opn*^-/-^ mice expressed keratin 14 and vWF after induction, with lower expression levels observed in the MSCs from the *Opn*^-/-^ mice. In vivo, MSCs could be recruited to wound sites and differentiate into multiple skin cell types including keratinocytes and endothelial cells, contributing to wound healing. More MSCs were recruited to the wound sites of the wild-type mice and exhibited higher differentiation efficiency compared to the *Opn*^-/-^ mice. In addition, MSCs treatment by intradermal injection significantly accelerated wound closure in wild-type mice, but no similar treatment effect was observed in the *Opn*^-/-^mice. Finally, the expression of the adhesion molecule, CD44, increased in the skin lesions of the wild-type mice, compared to the *Opn*^-/-^-mice. These data suggest that the OPN-CD44 pathway is involved in MSC-mediated skin wound healing.

Chronic or refractory skin wounds are an ongoing challenge in wound care. Transplanted-MSCs can differentiate into various cell lineages in wound sites and, provide immunomodulatory effects. Standal et al. demonstrated that OPN acted as a chemoattractant for a variety of cells types [39], but whether it has any role in MSCs in skin wound healing remains undetermined. In this paper, we showed that MSCs migrated to wound sites in an OPN-dependent manner. To further validate this finding, GFP-positive MSCs were intradermally injected around wounds and a stronger GFP signal was detected in the skin wounds of wild-type mice than in the skin wounds of *Opn*^-/-^ mice at day 3. These data confirm that OPN plays a critical role in the migration of MSCs to wounds.

This paper confirms that MSCs could differentiate into keratinocytes and endothelial cells both in vitro and in vivo. However, OPN deficiency decreases the differentiation efficiency of MSCs indicating that OPN could stimulate specific skin cell differentiation in MSCs.

Cell migration involves a cascade of events initiated by shear-resistant adhesive interactions between flowing cells and the vascular endothelium at the target tissue[40]. The selectin family mediates leukocyte tethering and rolling interactions on endothelial cells. This paper shows that *Opn*^*-/-*^ mice exhibit decreased CD44/E-selectin expression compared to wild-type mice, which may lower the efficiency of the tethering and rolling of MSCs on the endothelial surface. We speculate that OPN plays a key role in CD44/E-selectin-mediated MSCs rolling in the vascular endothelium.

Taken together, our data demonstrate that trauma results in the up-regulation of OPN, thereby facilitating the mobilization of MSCs for wound healing. In addition, accumulating MSCs can transdifferentiate into multiple skin cell types in the wound, thus contributing to repair.
